# Silicon electrowinning by molten salts electrolysis

**DOI:** 10.3389/fchem.2023.1133990

**Published:** 2023-02-02

**Authors:** Sai Krishna Padamata, Gudrun Saevarsdottir

**Affiliations:** ^1^ Department of Engineering, Reykjavik University, Reykjavik, Iceland; ^2^ Department of Materials Science and Engineering, NTNU, Trondheim, Norway

**Keywords:** silicon, molten salts, electrodeposition, electrolysis, Si films

## Abstract

Electrochemically produced Si in molten salts can be used to fabricate electronic and photovoltaic devices. The major factors influencing the structure and morphology of Si deposits are electrolyte composition, applied current densities and overpotentials, type of precursors, operating temperature, and electrodeposition duration. For Si electrodeposition, a less corrosive electrolyte with the ability to dissolve Si species and easily soluble in water should be used. This review provides a brief analysis of the Si production by electrolysis in molten salts.

## Introduction

Silicon is the second most abundant element in the earth’s crust, in the form of silica and metallic silicates (Greenwood and Earnshaw, 1997). Metallurgical-grade silicon (MG-Si) is produced using carbothermic reduction in a submerged arc furnace. In this process, the reduction of SiO_2_ by coke (elemental carbon or charcoal) at high process temperatures of 1,700°C or above and the following net reaction (**1**) takes place:

$$SiO_{2}\left(s\right)+C\left(s\right)\to Si\left(l\right)+CO_{2}\left(g\right)$$ (1)

The specific electric energy consumption is (11.5–13 kWh/kg Si), and carbon materials entering the process represent similar energy contributions. About half of that energy is retained as the chemical energy in Si metal. The carbon footprint ranges from 4.7 kg CO_2_/kg Si to 16 kg CO_2_/kg Si) depending on the type of energy sources used in the process (Xiao et al., 2010; Sævarsdottir et al., 2021). The purity of MG-Si produced from the carbothermic process is around 98% and 99%. Electronic-grade silicon (impurity content <1 PPB) and solar-grade silicon (impurity content <1 PPM) is used in various applications, such as in photovoltaics and electronics (Suzdaltsev, 2022). The conventional technique used to produce high-purity silicon from MG-Si is the Siemens process, which has high energy consumption and low productivity (Chigondo, 2018), or alternatively the fluidized bed process is used (Arastoopour et al., 2022).

An alternative method would be the electrodeposition of Si in molten salts, which is expected to produce high-purity silicon. If the anodes used are non-consumable and do not produce CO_2_, the carbon footprint can be significantly lowered compared to a conventional process, if the electricity used for electrolysis is renewable or nuclear. It has been proven that Si films with different morphologies can be electrochemically deposited in different molten salts such as chlorides, fluorides and chloride-fluorides (Juzeliu̅nas and Fray, 2020). Each of these salts has advantages and disadvantages; chloride melts are highly water-soluble, but Si films deposited are thin (<10 µm). Meanwhile, Si films deposited in fluorides are dense, but the salts adhering to the deposits cannot be easily removed.

Si can be deposited by adding Si source/precursors such as SiO_2_, Na_2_SiF_6_, K_2_SiF_6_, and SiCl_4_ to the molten salts. The Si precursors are decomposed to Si(IV) electroactive ions, which are reduced either by one-step or two-step reduction mechanisms based on the salt type. The structure and morphology of Si deposits can be controlled by varying the parameters, such as melt compositions, applied current densities, substrates and metallic dopants. The molten salt selected for Si electrodeposition should have either high solubility in an aqueous medium or high vapour pressure during high temperature distillation. The electrolysis process should be performed at low temperatures (≤850°C), have high current efficiency and be environmentally friendly. Sakanaka and Goto suggest that an inert anode should be used during the Si electrodeposition to avoid the evolution of CO_2_ and other carbon-containing gases (Sakanaka and Goto, 2015). This mini-review briefly discusses the reports on Si electrodeposition in chloride, fluoride and chloride-fluoride melts.

## Molten salts

### Chloride-based electrolytes

Chloride-based electrolytes have shown promising results in electrodepositing high-purity silicon. Some frequently studied chloride-based melts for Si deposition are CaCl_2_, KCl, NaCl, LiCl-KCl, and NaCl-KCl. Majority of the studies conducted to produce Si using SiO_2_ as a raw material used CaCl_2_-based electrolytes. CaCl_2_ melts have advantages such as high solubility in water (Garrett, 2004), low corrosivity (Janz and Tomkins, 1979) and high oxygen ions solubility (Wenz et al., 1969). Moreover, CaCl_2_ is non-toxic, inexpensive and widely available. Si electrodeposition is possible at a low temperature (500°C) in eutectic LiCl-KCl-CaCl_2_ melt (Yasuda et al., 2005a). The low-temperature electrodeposition process reduces energy consumption but lowers the process rate due to slower oxygen diffusivity in a solid phase.

Nohira et al. (2003) reported that the solid SiO_2_ can be electrochemically reduced to crystalline Si in CaCl_2_ molten salts. Electrons are transferred to the SiO_2_ electrode through a pinpoint electrical contact by conducting metal (Mo). When the potential applied is more negative than the reduction potential of SiO_2_, the electrochemical reaction occurs, and the following reaction is expected to take place:

$$SiO_{2}\left(s\right)+4e^{-}\to Si\left(s\right)+2O^{2-}$$ (2)

As the deoxygenation of SiO_2_ results in Si formation, the gaps formed between the Si atoms are filled with constituents of the molten salt. The new conducting medium is Si (transferring electrons to SiO_2_), which has good electrical conductivity (50 Ω^−1^cm^−1^at 850°C) at high temperatures. Three-phase interface between SiO_2_, molten salt and Si and continuous formation of Si takes place. Si formed is amorphous and transforms into crystalline Si at high temperatures through the bond-breaking process (Yasuda et al., 2005b).

**FIGURE 1:**
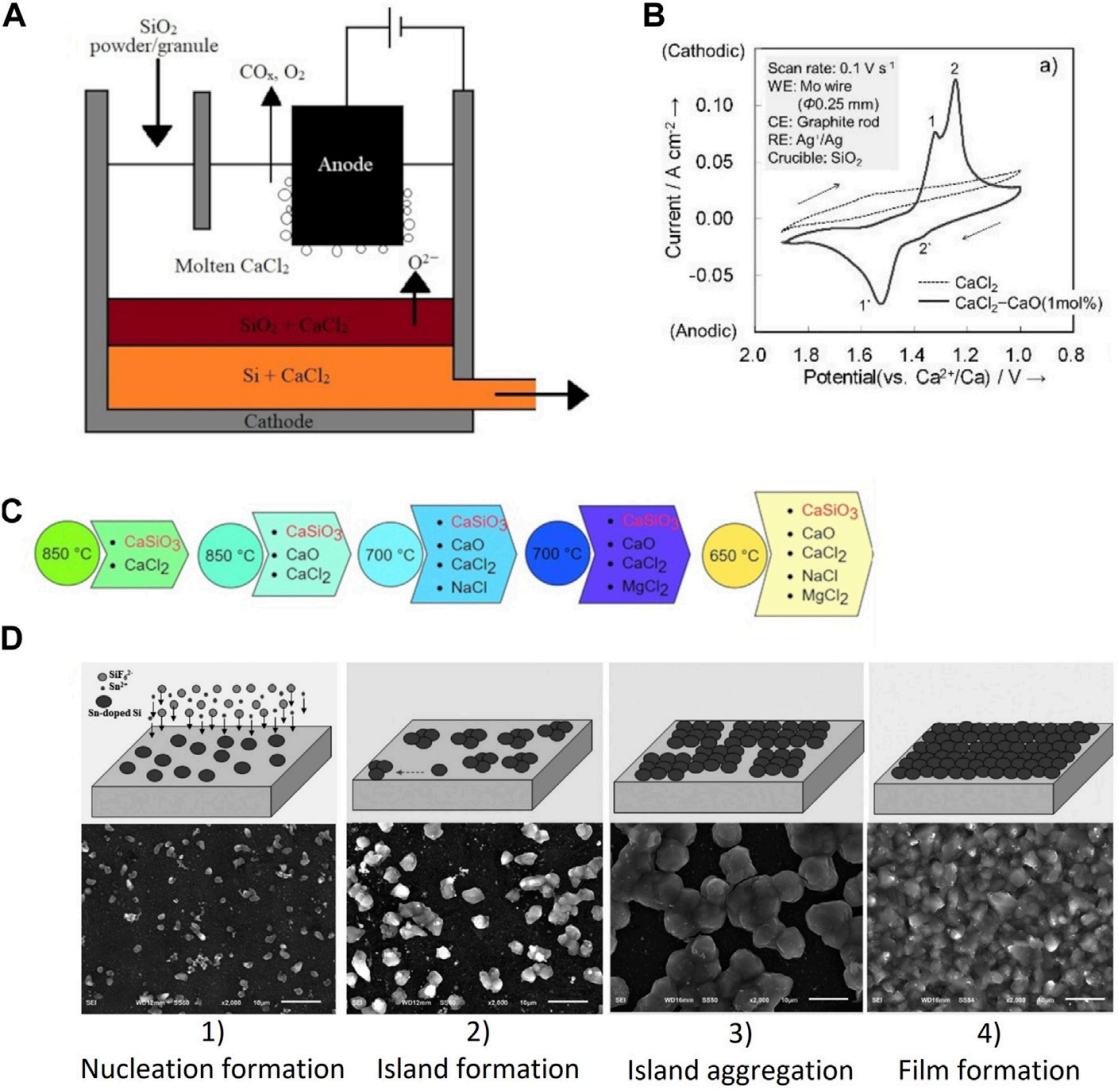
**(A)** The principle of the bottom cathode cell for the semi-continuous electrolysis of SiO_2_ powder (redrawn from Toba et al., 2013), **(B)** Cyclic voltammograms of a Mo wire in molten CaCl_2_ and CaCl_2_−CaO (1 mol%) in a SiO_2_ crucible at 1,123°C (reused with permission from Yang et al., 2017), **(C)** The progression of reaction designs that led to the final efficient low-temperature electrochemical reduction of CaSiO_3_ precursor (redrawn from Dong et al., 2017), **(D)** Schematic representation of growth process of tin-doped silicon film from electrodeposition on graphite foil substrate at 650°C in KCl–KF-1 mol% K_2_SiF_6_ salt with 0.02 wt% Sn dopant and corresponding morphologies of samples with different time at 5 mA cm^−2^: 1) nucleation, in 5 min; 2) island formation, in 15 min; 3) island aggregation, in 30 min; 4) film formation, more than 90 min (reused with permission from Peng et al., 2018).

Silicon electrodeposition can be achieved by reducing SiO_2_ nanoparticles (NP) in CaCl_2_ molten salts (Cho et al., 2012). Silicon layers were fabricated on Mo substrate in molten salts by directly adding and dissolving SiO_2_ NP at 850°C. The deposited Si layer had high purity and was crystalline, but the deposited Si was not a continuous film. Toba et al. reported the direct electrolytic reduction of SiO_2_ in molten CaCl_2_ at 850°C to produce SOG-Si (Toba et al., 2013). The concept they proposed is similar to the Hall-Heroult process for aluminium production. Granular/powder SiO_2_ is electrochemically reduced to Si, deposited at the cathode, and slurry containing Si and molten CaCl_2_ is continuously tapped (Figure 1A). Crystalline Si was produced with applied potentials between 0.5 and 0.7 V (vs. Ca^2+^/Ca). During the electrolysis with potentials 0.5 and 0.6 V, the rate-determining step was the diffusion of oxygen ions through CaCl_2_ melt in the produced Si layer on the unreduced SiO_2_ core. Experimental results suggest that it is possible to deposit a continuous SOG-Si layer in CaCl_2_ melt. During CaCl_2_-SiO_2_ electrolysis with a graphite foil cathode and graphite anode, the cell voltage determines the morphology of the Si deposited (Xie et al., 2018). For instance, at cell voltage below 2.0 V, Si nanowires with diameters between 50 and 100 nm are prepared. Under 2.2 V, Si nanoparticles are deposited on substrates and between 2.4 and 2.6 V, a dense Si film is deposited. In addition to cell voltage, the substrate also plays a vital role in depositing Si films. A dense Si film (a thickness of 3.5 µm) is deposited on a graphite substrate with silicon carbide acting as an interface between the Si film and substrate. Meanwhile, a porous (a thickness of 7 µm) Si film is deposited on the Ag substrate, resulting in the crushing of Si films due to porosity (Zhao et al., 2016).

The Si electrodeposition can be enhanced by adding CaO as a dopant to the molten CaCl_2_ (Yang et al., 2017). The cyclic voltammetry curves confirm that the cathode peak associated with Si reduction intensifies with the addition of CaO 1 mol% (Figure 1B). Further, a dense crystalline layer of Si with a thickness of 35 µm was successfully deposited on a graphite substrate during potentiostatic electrolysis at 0.7 V. Optimal electrolyte composition for Si electrodeposition was reported as CaCl_2_−(4.8 mol%) CaO−(3.9 mol%) SiO_2_. The addition of CaO can accelerate the Si electrodeposition as CaO helps in the continuous ionisation of silicon dioxide to form silicate ions (SiO_3_^2−^, SiO_4_^4−^, etc.) (Zou et al., 2019; Zou et al., 2020; Li et al., 2022). Zou et al. deposited Si films with a purity of 99.99989 in CaCl-CaO-SiO_2_ melt (Zou et al., 2019). The presence of CaO enables the following reactions (3–5):

$$xSiO_{2}+yCaO\left(Ca^{2+},O^{2-}\right)\;\to Ca_{y}Si_{x}O_{2x+y}\left(yCa^{2+},Si_{x}O_{2x+y}^{2y-}\right)$$ (3)

$$Si_{x}O_{2x+y}^{2y-}+4xe^{-}\to xSi+\left(2x+y\right)O^{2-}$$ (4)

$$xS{iO}_{2}+yO^{2-}\to Si_{x}O_{2x+y}^{2y-}$$ (5)

The melting point of pure CaCl_2_ is 772°C, and electrolysis at such high temperatures would need more energy, resulting in corrosion of the electrolysis cell and thermal shock of electrodes. Including other chloride salts in the CaCl_2_ melt could substantially reduce the liquidus temperature of the mixture. For instance, Sakanaka et al. (2017) reported electrolytic deposition of Si on Mo substrate in a eutectic BaCl_2_-CaCl_2_-NaCl-SiO_2_ mixture at 650°C. For 1 h of potentiostatic electrolysis at −1.60 V, a dense Si film was deposited with a thickness of 1 µm. However, with an increase in electrolysis time to 3 h, a porous and less crystalline Si layer was obtained. The powdery and dendritic deposits with prolonged electrolysis time as some of the deposited Si interacts with Si(IV) and forms divalent silicon ions, which can be ascribed to the following reactions (6, 7):

$$Si\left(IV\right)+4e^{-}\to Si$$ (6)

$$Si+Si\left(IV\right)\to 2Si\left(II\right)$$ (7)

At low temperatures, SiO_2_ has low solubility in chloride melts which would affect the morphology of Si films. CaSiO_3_ could be used as an alternative Si precursor in low-temperature chloride melts (Figure 1C). The CaSiO_3_ easily dissolves in this chloride-based melt and generates Ca^2+^ and SiO_3_^2−^ ions. Dong et al. reported electrolytic production of Si nanowires in CaCl_2_-MgCl_2_-NaCl-CaO using CaSiO_3_ as raw material at 650°C (Dong et al., 2017). The Si nanowires production had a high yield and were crystalline but only had a purity of 99.58%, which is metallurgical grade.

Si nanotubes can be electrodeposited by co-reduction of SiO_2_ and AgCl on Ni substrate in NaCl-CaCl_2_ (Weng et al., 2020). Initially deposited Ag on the Ni will facilitate the formation of liquid Ag-Si intermetallic, promoting Si nanowires’ growth. Ag and Si are separated during the cooling process, where Ag is deposited on Ni. Si nanowires can also be electrodeposited in KCl-containing K_2_SiF_6_ at temperatures below 800°C (Trofimov et al., 2022). The morphology of the Si nanowires highly depends on the overpotential applied during the potentiostatic electrolysis. Adding CsCl to molten KCl-containing K_2_SiF_6_ can reduce the liquidus temperature of the melt and the diameter of Si nanowires. The decrease in cathodic current density would increase the diameter of Si nanowires (Gevel et al., 2022a). In KCl-K_2_SiF_6_ melts, the Si(IV) reduction process comprises a reversible 4-electron process, Si nanofibers with a diameter of 200 to 300 nm and a purity of 99.99% can be deposited (Gevel et al., 2022b). The main advantage of using the KCl-K_2_SiF_6_ system is that the anionic composition does not change. That results in the stability in energy characteristics of Si-containing electroactive ions and control of the morphology of Si deposits (Gevel et al., 2021).

### Fluoride-based electrolytes

The Si electrodeposition in various molten fluorides, like LiF-KF (Cohen and Huggins, 1976; Rao et al., 1981) and LiF-NaF-KF (Elwell and Rao, 1982) containing K_2_SiF_6_ precursor, has been studied since the 1970s. One major advantage of fluoride melts is the high solubility of SiO_2_ or other Si precursors, enabling the high thickness of deposited Si films. For instance, Si film deposited by galvanostatic electrolysis for 3 h at 40 mAcm^−2^ and 800°C in eutectic LiF-KF melt with K_2_SiF_6_ (5 mol%) has a thickness of 150 µm (Haarberg et al., 2013). The Si deposition was a two-step process, i.e., Si(IV)→Si(II)→Si. A loss of current efficiency (ranging from 85% to 95%) is related to the existence of Si(II) (reaction 6). Similar results were reported by Boen and Bouteillon, where the Si depositions involve Si(IV) and Si(II) species in LiF-NaF-KF melts containing Na_2_AlF_6_ (Boen and Bouteillon, 1983).

Bieber et al. reported that of all the eutectic fluoride melts containing SiF_4_(g), Si(IV) has the best stability in the NaF-KF mixture, and Si(IV) volatility increases with an increase in the acidic nature (most basic to most acidic: NaF–KF < LiF–KF < NaF–MgF_2_ < NaF–CaF_2_ < LiF–NaF < LiF < LiF–CaF_2_) of the melt (Bieber et al., 2011). Eutectic NaF-KF melts containing Na_2_SiF_6_ have been used in Si electrodeposition on various substrates (Ag, Ni, and C) and operating conditions (Bieber et al., 2012). Out of films formed on Ag, Ni, and C, Si films deposited on C were the most coherent and had the highest purity, and a SiC interface was observed between the Si film and C. At high current densities, the limiting diffusion rate of Si-ions strongly influences the Si film growth, leading to dendritic and rough deposits.

The solubility of SiO_2_ is enhanced by adding O^2−^ ions in the fluoride melts, similar to that of chloride melts. Suzuki et al. (2019) investigated the influence of Li_2_O addition to KF, LiF-KF, and LiF-NaF-KF molten salts on SiO_2_ electroreduction . Compared to Si films deposited in melts without LiO_2_, Si films deposited in melts containing Li_2_O were around 6 to 23 times thicker and were obtained with better current efficiency, depending on the applied potential during the electrodeposition. The phenomenon is due to the capability of O^2−^ (sourced through Li_2_O) to break the Si-O-Si bond of SiO_2_ and complex silicate ions (e.g., Si_2_O_5_^2−^) to promote the formation of easily reduced silicate ions (e.g., SiO_3_F^3−^). The presence of multiple Si ions combined with fluoride, oxyfluorides, and oxides would negatively affect the current efficiency of Si deposition. For instance, Sakanaka and Goto (2015) electrodeposited Si films in eutectic NaF-LiF-KF melt with a current efficiency of 10%. Si(IV) ions in multiple forms would have different reduction potentials, and when broader potentials are applied for electroreduction, alkali metals tend to co-deposit along with Si.

In BaF_2_-CaF_2_-SiO_2_ melts, the electrodeposition of Si on Mo substrate was a one-step process, i.e. Si(IV)→Si (Hu et al., 2013). SEM examination of the deposits after 8 h of galvanostatic polarisation at −0.118 Acm^−2^ revealed that an intermetallic phase MoSi_2_ (thickness <10 µm) was formed, which acted as an interface between the Si layer (thickness >50 µm) and the Mo substrate. Even though a thick Si film is deposited in this melt, the process is performed at very high temperatures (1,300°C), which requires a lot of energy and harms the electrolysis setup.

### Chloride-fluoride-based electrolytes

For Si electrodeposition, molten chloride-fluoride salts are better electrolytes than individual chloride and fluoride melts. It is because the Si films deposited in chloride melts have a thickness of less than 10 µm and the solubility of Si species is low. On the other hand, fluoride melts are not water soluble, and the salts adhering to the Si films are difficult to remove. Moreover, molten fluorides are corrosive and could damage the reactor walls (Xu and Haarberg, 2013). KF-KCl mixtures are considered promising electrolytes for Si electrodeposition as the mixture has sufficient thermal stability, is less aggressive than molten fluorides, is water-soluble (until KF mol% is 66%) and has good Si species solubility at low temperatures (at 650°C) (Zaikov et al., 2013; Maeda et al., 2014; Maeda et al., 2015).

Si(IV) reduction is a one-step process in KF-KCl-K_2_SiF_6_ melt, where the Si(IV) reduction is governed by a quasi-reversible-reversible electron transfer reactions mechanism (Maeda et al., 2015). Zhuk et al. (2017) deposited a crystalline silicon film (purity 99.9%) on Ag, C, and Ni substrates in KF-KCl melts containing K_2_SiF_6_. The authors confirm that SiC was not formed on graphite during the Si deposition, whereas the presence of SiC (thickness of 10 µm) intermetallics was reported by Bieber et al. (2012). Adding KI to KF-KCl melt would reduce the melt’s aggressiveness and liquidus temperature but decrease the electrical conductivity (Laptev et al., 2021). However, the reduced electrical conductivity would contribute to forming more compact and adhesive Si films (Laptev et al., 2020). It has been stated that during the electrodeposition on energetically non-homogeneous surfaces, an increase in the specific electrical resistance of the melt can cause the smoothing effect at the electrodeposition from the melt (Laptev et al., 2020).

The semiconductor characteristics of Si films deposited can be modified based on the Si precursor metallic dopant added to the melt. For instance, Si films electrodeposited in KF–KCl–K_2_SiF_6_ molten salts exhibit n-type semiconductor characteristics, while Si films deposited in KF–KCl–SiCl_4_ melts exhibit p-type semiconductor characteristics (Yasuda et al., 2021). The difference in the semiconductor characteristics can be attributed to the different impurities present in the Si films. Both Si films had a purity of 99.99% (4 N). A dense crystalline Si film (thickness up to 60 µm) has been deposited in KF-KCl-K_2_SiF_6_ melts containing Sn-dopant (0.020–0.035 wt%) (Peng et al., 2018). Co-deposited Sn along with Si could induce the lateral growth of Si thin films. A four-step mechanism is involved in the Si film formation: nucleation, island formation, island aggregation and film formation (Figure 1D). The tin-assisted Si film deposits show n-type semiconductor characteristics.

Li et al. (2018) investigated the electrochemical behaviour of Si(IV) on W and Mo (Li et al., 2019) electrodes in CaCl_2_-CaF_2_-CaO-SiO_2_ melts and found that on both the electrodes Si(IV) reduction involves one-step reduction mechanism and follows an instantaneous nucleation mode. MoSi_2_ intermetallic phase is formed when the Si films are deposited on the Mo electrode (Li et al., 2019), similar to what Hu et al. obtained during the electrodeposition in fluoride salts (Hu et al., 2013).

The morphology of Si films also depends on the type of alkali melt used during the electrolysis. Norikawa et al. (2022) studied the electrochemical behaviour of Si(IV) in MF-MCl-M_2_SiF_6_ melts (where M = Li, Na, K, Cs) at 800°C. For Li-systems, it was noted that no peaks related to Si were observed during the cyclic voltammetry, which is attributed to the formation of Li-Si intermetallics. In Na-systems, whisker-like Si films were deposited, while in K and Cs systems, smooth and adherent films were deposited on Ag substrates. Further examination using short-term galvanostatic polarisation revealed that the cathodic potential rapidly changed in Na-system. At the same time, the cathodic potential remained constant in K-systems, which means side reactions are taking place in Na-system.

## Concluding remarks

Si with different types of structures and purity levels can be readily produced by electrochemical deposition in molten salts. The most extensively studied melts are CaCl_2_-CaO-SiO_2_/CaSiO_3_ and KF-KCl-K_2_SiF_6_ mixtures. In chloride-based melts, a high-purity Si can be deposited on substrates. The CaCl_2-_based melts are highly water soluble, but the thickness of the Si films remains less than 10 µm. Si films deposited in KF-KCl melts are dense and smooth with thickness above 20 µm. However, the purity of Si films is only limited to 99.99%. The one-step reduction of Si(IV) is preferred as the existence of Si(II) in the system would reduce the current efficiency and result in the deposition of non-coherent Si films.

An ideal electrolyte should readily dissolve Si compounds, have a high reaction rate, the Si films deposited on the dense and non-porous and the electrolytes should be highly soluble in water. Moreover, the electrolyte should have a low liquidus temperature so the electrolysis cell can have a longer lifespan and process requires less energy. Chloride-fluoride-based melts have the combined advantages of fluoride and chloride melts, such as dense and thick Si film formation, which can easily dissolve Si precursors and have a low-liquidus temperature. In particular, KF-KCl has high water solubility, and dense Si films can be deposited in this system. Si electrodeposition in KF-KCl melts in a one-step process. Using inert anodes instead of carbon anodes for Si electrowinning would avoid the evolution of CO_2_. However, no significant efforts have been made to develop an inert anode system for Si electrodeposition. One of the prerequisites to adopting inert anodes is to have low-temperature melts so that anodes can have a longer lifetime. This would be one more reason to use KF-KCl melts, as the eutectic temperature of this mixture is 605°C.
